# Beyond Rhythm Control: Health-Related Quality of Life and Attention–Behavioral Outcomes After Catheter Ablation in Children with Supraventricular Tachycardia

**DOI:** 10.3390/healthcare14070898

**Published:** 2026-03-31

**Authors:** Tuğba Doğaç, Özlem Elkıran, Serdar A. Maraş, Mehmet Öncül, Özlem Doğan

**Affiliations:** 1 Faculty of Medicine, Malatya Turgut Özal University, 44000 Malatya, Turkey; 2 Faculty of Medicine, Inonu University, 44280 Malatya, Turkey; 3 Malatya Training and Research Hospital, 44000 Malatya, Turkey

**Keywords:** pediatric supraventricular tachycardia, catheter ablation, health-related quality of life, attention and behavioral outcomes, pediatric arrhythmia, neurobehavioral functioning, child and adolescent psychiatry

## Abstract

**Background:** Supraventricular tachycardia (SVT) is the most common symptomatic arrhythmia in childhood and may negatively affect physical, emotional, and psychosocial functioning. Although catheter ablation is an established treatment for SVT, most outcome studies have focused primarily on rhythm control rather than broader psychosocial recovery. **Objective:** This study aimed to evaluate changes in health-related quality of life (HRQoL) following catheter ablation in children with symptomatic SVT and to characterize attention–behavioral outcomes at follow-up. **Methods:** In this retrospective–prospective observational cohort study, pediatric patients aged 4–17 years who underwent successful catheter ablation between January 2022 and June 2025 were included. HRQoL was assessed using the Pediatric Quality of Life Inventory (PedsQL™), while psychiatric diagnoses were determined using the Schedule for Affective Disorders and Schizophrenia for School-Age Children-Present and Lifetime Version (K-SADS-PL) interview and attention–behavioral symptoms were evaluated using the Disruptive Behavior Disorders Scale (DBDS). **Results:** A total of 59 patients (median age 13 years; 57.6% girls) were included. Significant improvements were observed across all HRQoL domains after ablation (all *p* < 0.001), with large effect sizes indicating substantial gains in patient-reported functioning. At follow-up, 30.5% of participants met criteria for at least one psychiatric diagnosis, most commonly anxiety disorders. Boys demonstrated significantly higher inattention scores than girls (*p* = 0.008), and age was positively associated with inattention and conduct-related symptoms. **Conclusions:** Catheter ablation for pediatric SVT was associated with marked improvements in HRQoL, supporting its benefits beyond rhythm control. However, a meaningful proportion of patients exhibited psychiatric or attention–behavioral symptoms at follow-up. These findings highlight the importance of integrating psychosocial assessment into the clinical follow-up of children undergoing arrhythmia treatment.

## 1. Introduction

Pediatric cardiac arrhythmias, although less common than in adults, may substantially affect children during critical periods of physical, cognitive, and psychosocial development. Among these conditions, supraventricular tachycardia (SVT) is the most common symptomatic arrhythmia in childhood and adolescence [1,2]. Epidemiological studies estimate that SVT occurs in approximately 1 in 250–1000 children depending on the population studied and the diagnostic criteria applied [1]. Recurrent tachycardia episodes may lead to palpitations, fatigue, exercise intolerance, anxiety, and school absenteeism, thereby negatively affecting daily functioning and psychosocial well-being in affected children and their families [2,3].

Catheter ablation has become an established and highly effective treatment for recurrent or symptomatic SVT in pediatric populations. Over the past decade, advances in electrophysiological techniques—including three-dimensional electroanatomic mapping systems, improved catheter technologies, and fluoroscopy-reduction strategies—have significantly improved procedural success rates while minimizing complications and radiation exposure [4,5,6]. Contemporary consensus guidelines from pediatric electrophysiology societies recognize catheter ablation as a definitive therapeutic option for many children with symptomatic SVT, particularly when symptoms persist despite pharmacological therapy or when antiarrhythmic medications are poorly tolerated [6].

Recent studies in pediatric electrophysiology have continued to report high procedural success rates exceeding 90–95% with low complication rates for catheter ablation procedures in children and adolescents [7,8]. These advances have strengthened the role of catheter ablation as a definitive therapeutic option for symptomatic SVT in pediatric populations.

Beyond arrhythmia elimination, successful catheter ablation may have broader implications for patient well-being. In recent years, health-related quality of life (HRQoL) has emerged as an important patient-centered outcome in pediatric cardiology research. HRQoL reflects multiple domains of functioning, including physical health, emotional well-being, social participation, and school performance [9]. Previous studies have shown that children with symptomatic SVT often experience impaired HRQoL compared with healthy peers and that quality of life may improve following successful catheter ablation [10,11]. These findings suggest that recurrent arrhythmia symptoms may substantially influence daily functioning and psychosocial adjustment in pediatric patients.

Despite these advances, outcome assessments following pediatric catheter ablation have traditionally focused on electrophysiological success and arrhythmia recurrence. Comparatively less attention has been given to neurobehavioral and psychological outcomes, which represent important aspects of child development and long-term well-being. This gap is clinically relevant because children with chronic medical conditions have been shown to exhibit higher rates of emotional and behavioral difficulties compared with healthy peers [12]. Illness-related stress, activity limitations, repeated healthcare encounters, and uncertainty related to symptoms may contribute to anxiety, attentional difficulties, and behavioral challenges during childhood and adolescence [12,13].

Children with recurrent arrhythmias may be particularly vulnerable to such psychosocial challenges. Anxiety associated with unpredictable tachycardia episodes, sleep disturbances, reduced participation in physical activities, and prolonged symptom exposure prior to definitive treatment may influence emotional regulation and attentional functioning [13,14]. However, empirical data regarding behavioral and psychiatric outcomes following catheter ablation in pediatric populations remain limited.

To our knowledge, few studies have simultaneously evaluated HRQoL together with structured psychiatric assessment and attention–behavioral functioning in children undergoing catheter ablation for supraventricular tachycardia.

Therefore, the present study aimed to (i) evaluate changes in health-related quality of life before and after catheter ablation in children with symptomatic supraventricular tachycardia and (ii) characterize attention–behavioral functioning approximately six months after ablation, with particular attention to age- and sex-related patterns.

## 2. Materials and Methods

### 2.1. Study Design, Setting, and Reporting Standard

This study was designed as a retrospective–prospective observational cohort study and was conducted at the Pediatric Cardiology Clinic of İnönü University, a tertiary referral center for pediatric arrhythmia management. Pediatric patients who underwent catheter ablation for symptomatic supraventricular tachycardia (SVT) between January 2022 and June 2025 were retrospectively identified from institutional electrophysiology and ablation records.

Retrospective data collection included demographic characteristics, clinical and ablation-related variables, and baseline health-related quality of life (HRQoL) data. Prospective data collection included post-ablation HRQoL assessment, structured psychiatric interview, and attention–behavioral symptom assessment performed during follow-up visits.

**Figure 1:**
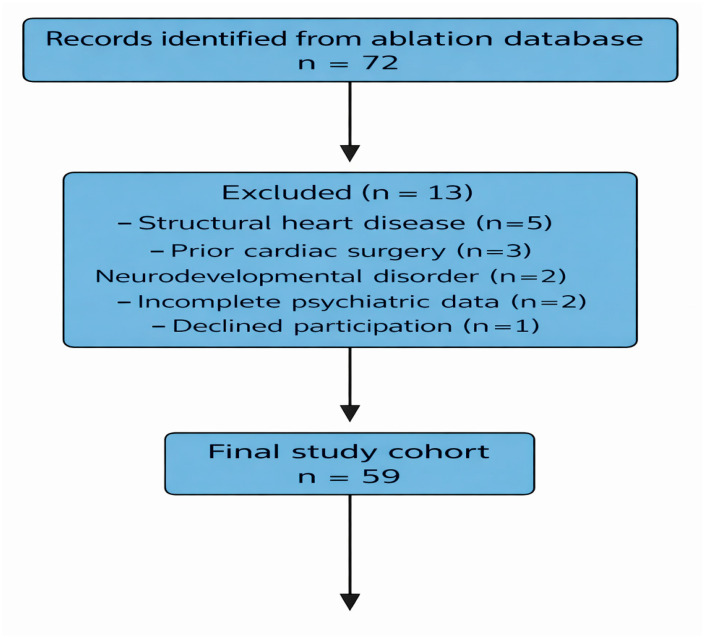
Strengthening the Reporting of Observational Studies in Epidemiology (STROBE) compliant flow diagram illustrating patient identification, eligibility assessment, exclusions, and final inclusion in the study cohort.

The study was reported in accordance with the Strengthening the Reporting of Observational Studies in Epidemiology (STROBE) guidelines for cohort studies. Participant selection, exclusions, non-participation, and final inclusion are illustrated in a STROBE-compliant flow diagram (Figure 1). A consecutive sampling strategy was used, and all eligible patients with acute procedural success were contacted consecutively for follow-up assessment.

Because the study was conducted at a single tertiary pediatric cardiology center and the final analytical sample was restricted to patients with successful acute ablation, the findings may not be fully generalizable to all pediatric SVT populations.

### 2.2. Ethical Approval

The study protocol was approved by the Malatya Turgut Özal University Clinical Research Ethics Committee (approval number: 2025/394; 16 October 2025). Institutional permission was obtained from İnönü University Faculty of Medicine Hospital. Written informed consent was obtained from parents or legal guardians, and assent was obtained from children when developmentally appropriate. All procedures were conducted in accordance with the Declaration of Helsinki.

### 2.3. Study Population and Eligibility Criteria

The study included 59 children and adolescents aged 4–17 years who underwent catheter ablation for symptomatic SVT and completed post-ablation follow-up evaluation. The final sample consisted of 34 girls (57.6%) and 25 boys (42.4%).

#### 2.3.1. Inclusion Criteria

Participants were included if they met all of the following criteria:

(i) age between 4 and 17 years;
(ii) diagnosis of symptomatic supraventricular tachycardia;
(iii) successful catheter ablation performed during the study period; and
(iv) availability for post-ablation psychiatric evaluation and HRQoL assessment.

#### 2.3.2. Exclusion Criteria

Participants were excluded if they had:

(i) structural or congenital heart disease;
(ii) history of cardiac surgery;
(iii) known neurological or neurodevelopmental disorders;
(iv) known genetic syndromes;
(v) incomplete psychiatric assessment data; or
(vi) refusal to participate.

Patients with asymptomatic pre-excitation (e.g., Wolff–Parkinson–White pattern without documented tachyarrhythmia or clinical symptoms) were not included, as the study focused exclusively on symptomatic SVT with a clear clinical indication for catheter ablation.

Successful ablation was defined as complete elimination of the arrhythmogenic substrate with no recurrence of arrhythmia during the acute post-procedural period. Because the study was designed to evaluate HRQoL and psychiatric outcomes after successful rhythm control, analyses were restricted to patients with acute procedural success in order to reduce confounding from persistent arrhythmia or immediate procedural failure. The potential selection bias introduced by this restriction is acknowledged in the Discussion.

### 2.4. Catheter Ablation Procedure and Definition of Success

All catheter ablation procedures were performed by experienced pediatric electrophysiologists according to contemporary pediatric electrophysiology practice. Procedures were performed under conscious sedation or general anesthesia in accordance with institutional protocols.

Three-dimensional electroanatomic mapping systems were routinely used to guide catheter positioning and minimize fluoroscopy exposure, including the EnSite Precision system (Abott Laboratories, Abbott Park, IL, USA). Radiofrequency or cryoablation energy sources were applied depending on arrhythmia mechanism and anatomical considerations.

Procedural success was defined as complete elimination of the arrhythmogenic substrate without acute recurrence of tachyarrhythmia at the end of the electrophysiological study. Analyses were restricted to patients with acute procedural success.

Acute procedural complications were reviewed from routine institutional records, and no acute procedural complications were identified in the study cohort. Detailed procedural variables, including arrhythmia mechanism subtype (e.g., atrioventricular nodal reentrant tachycardia(AVNRT), atrioventricular reentrant tachycardia (AVRT), ectopic atrial tachycardia) ablation laterality, energy source distribution, fluoroscopy time, six-month recurrence status, and antiarrhythmic medication use at follow-up, were not systematically available for all participants and therefore were not included in the primary analysis.

### 2.5. Timing of Outcome Assessments 

Baseline health-related quality of life (HRQoL) referred to the month preceding catheter ablation. Because standardized HRQoL assessments were not routinely performed before the procedure in routine clinical practise, baseline HRQoL data were obtained during the follow-up visit using the Pediatric Quality of Life Inventory (PedsQL™ 4.0; Mapi Research Trust, Lyon, France; https://www.pedsql.org) based on caregiver recall. Caregivers were instructed to answer the questionnaire with reference to the one-month period preceding the ablation procedure.

To minimize recall bias, caregivers were provided with structured prompts and were asked to anchor their responses to the defined pre-ablation time window. Nevertheless, because baseline HRQoL data were collected retrospectively through caregiver recall, the possibility of recall bias cannot be excluded.

Post-ablation HRQoL assessment, psychiatric evaluation, and attention–behavioral symptom assessment were conducted during a scheduled outpatient follow-up visit approximately six months after catheter ablation. This follow-up interval was selected because it reflects a typical short-term clinical reassessment period in pediatric electrophysiology practice and allows sufficient time for early recovery, return to daily activities, and stabilization of symptom burden after ablation.

Psychiatric and behavioral assessments were obtained only at this post-ablation time point; no standardized pre-ablation psychiatric interview or attention–behavioral scale data were available in routine clinical records. Therefore, HRQoL was evaluated longitudinally (pre- vs. post-ablation), whereas psychiatric and attention–behavioral outcomes were characterized cross-sectionally at follow-up only.

**Figure 2:**
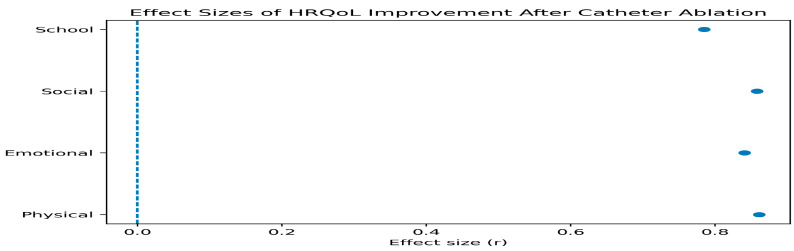
Timeline of catheter ablation and outcome assessments. Baseline health related quality of life (HRQoL) was assesed in the month preceding ablation, whereas psychiatric and behavioral assessments were conducted approximately six months after the procedure. Blue dashed lines indicate the timing of outcome assessments.

The study timeline is illustrated in Figure 2. 

### 2.6. Health-Related Quality of Life Assessment

Health-related quality of life was assessed using the Pediatric Quality of Life Inventory (PedsQL™ 4.0), a widely used and validated instrument for measuring HRQoL in pediatric populations. The instrument evaluates four domains of functioning: physical, emotional, social, and school functioning.

At the follow-up assessment, parent-proxy reports were used for children aged 4–7 years, whereas child self-report forms were used for participants aged 8–17 years in accordance with the instrument guidelines and developmental recommendations.

Because baseline HRQoL data were obtained retrospectively through caregiver recall, the baseline assessment for all participants was based on parent-reported information referring to the pre-ablation period. Follow-up assessments for older children and adolescents were based on child self-report when applicable. Consequently, an informant shift between baseline and follow-up assessments may have occurred in some cases. This methodological issue was considered when interpreting the HRQoL comparisons.

All PedsQL™ items were reverse-scored and linearly transformed to a 0–100 scale, with higher scores indicating better health-related quality of life. Subscale scores (Physical Functioning, Emotional Functioning, Social Functioning, and School Functioning) were calculated as the mean of completed items within each domain. The total HRQoL score was calculated as the mean of all completed items when at least 50% of items were available. No imputation procedures were applied for missing items.

Previous validation studies of the PedsQL™ have demonstrated acceptable to excellent internal consistency in pediatric populations for both child self-report and parent-proxy forms.

### 2.7. Psychiatric Assessment and Structured Clinical Interview

Psychiatric evaluation was conducted during a follow-up visit approximately six months after catheter ablation by a board-certified child and adolescent psychiatrist experienced in pediatric psychosomatic assessment. Psychiatric diagnoses were established according to the Diagnostic and Statistical Manual of Mental Disorders, Fifth Edition (DSM-5) [15].

For diagnostic assessment, the Schedule for Affective Disorders and Schizophrenia for School-Age Children—Present and Lifetime Version (K-SADS-PL) was used as the primary diagnostic instrument. Interviews were conducted in a semi-structured format in accordance with standardized administration guidelines and included both parent and child interviews when developmentally appropriate.

To ensure diagnostic consistency, all psychiatric interviews were performed by the same clinician using a standardized assessment protocol. Because of the observational design and the use of a single assessor, blinding to cardiology-related clinical information was not feasible. However, psychiatric evaluations were conducted independently of routine cardiology follow-up visits.

Previous psychiatric history, outpatient psychiatric follow-up, and current psychotropic medication use were assessed using caregiver reports and medical record review. Ongoing psychiatric treatment and medication use were not exclusion criteria and were recorded for descriptive purposes. Psychiatric diagnoses identified at follow-up were interpreted as concurrent post-ablation clinical findings rather than as incident outcomes attributable to catheter ablation.

### 2.8. Attention–Behavioral Symptom Assessment

Attention and behavioral functioning were assessed at follow-up using the DSM-IV–Based Disruptive Behavior Disorders Screening and Rating Scale (DBDS), a parent-report instrument evaluating symptoms across four domains: inattention, hyperactivity, oppositional defiant behaviors, and conduct-related problems. The DBDS was administered as a parent-report measure across the full study age range at follow-up.

DBDS assessments were performed only at the post-ablation follow-up visit; no baseline DBDS data were available before the procedure. Accordingly, DBDS results were used to characterize post-ablation attention–behavioral symptom burden and were not interpreted as pre–post change attributable to catheter ablation.

For descriptive purposes, predefined cutoff values were applied as follows: inattention ≥ 10, hyperactivity ≥ 10, oppositional defiant behaviors ≥ 6, and conduct-related problems ≥ 6. These thresholds correspond to symptom counts consistent with DSM-based screening approaches and have been used in prior validation and clinical research involving the Disruptive Behavior Disorders Rating Scale [16].

The Turkish version of the DBDS has been reported to have acceptable psychometric properties in prior studies. However, because complete item-level data were not available in a format suitable for all retrospective reliability analyses, internal consistency coefficients for the present cohort were not recalculated for all measures and time points.

### 2.9. PedsQL™ Scoring Procedure

All PedsQL™ items were scored according to the standard scoring protocol recommended by the instrument developers. Item responses were reverse-scored and transformed linearly to a 0–100 scale. Higher scores indicated better health-related quality of life.

Subscale scores were calculated as the mean of the relevant domain items. The total HRQoL score was computed as the mean of all answered items, provided that at least 50% of items were completed. No imputation methods were used for missing data.

All HRQoL outcomes were presented and analyzed using the standardized 0–100 scoring system.

### 2.10. Procedure

All psychiatric interviews and HRQoL assessments were conducted during a scheduled outpatient follow-up visit approximately six months after catheter ablation. Sociodemographic and clinical data were obtained from medical records and structured caregiver interviews.

Assessments were administered in a quiet outpatient setting following standardized administration procedures. Parent-proxy and child self-report forms were completed independently, thereby minimizing the potential influence of mutual interaction on assessment outcomes.

### 2.11. Selection Bias and Patient Flow

Because only patients who underwent successful catheter ablation and were available for follow-up were included, the possibility of selection bias was considered. To transparently present the process by which the final study sample was formed, patient flow from the initial ablation cohort to the final analytical sample is illustrated in the STROBE-compliant flow diagram (Figure 1).

Patients with unsuccessful ablation, incomplete follow-up, exclusion criteria, or refusal to participate were not included in the final cohort. This approach was adopted to improve internal consistency of the clinical sample but may have limited generalizability.

### 2.12. Statistical Analysis

Statistical analyses were performed using IBM SPSS Statistics version 26.0 (IBM Corp., Armonk, NY, USA). Continuous variables were summarized according to data distribution as mean ± standard deviation or median (interquartile range). Categorical variables were presented as frequencies and percentages.

Pre- and post-ablation HRQoL outcomes were compared using the Wilcoxon signed-rank test because of the non-normal distribution of PedsQL™ subscale scores. To account for multiple comparisons across the four HRQoL domains, Bonferroni correction was applied, with statistical significance defined as *p* < 0.0125. Effect sizes for these comparisons were calculated as r = |Z|/√N.

Sex-based comparisons of DBDS subscale scores were performed using the Mann–Whitney U test. Associations between age and DBDS subscale scores were examined using Spearman rank correlation coefficients. Proportions of participants exceeding predefined DBDS cutoff values were compared between sexes using chi-square or Fisher’s exact test, as appropriate.

No formal correction for multiple comparisons was applied to the exploratory DBDS subgroup and correlation analyses. Therefore, these analyses were interpreted cautiously, and the increased risk of type I error is acknowledged in the Discussion.

**Figure 3:**
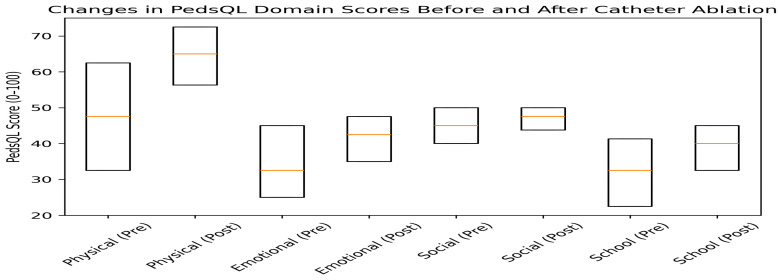
Changes in Pediatric Quality of Life Inventory (PedsQL™) domain scores before and after catheter ablation. Box plots illustrate median values, interquartile ranges, and distribution of health-related quality of life (HRQoL) scores across domains. The orange lines indicate the median values.

**Figure 4:**
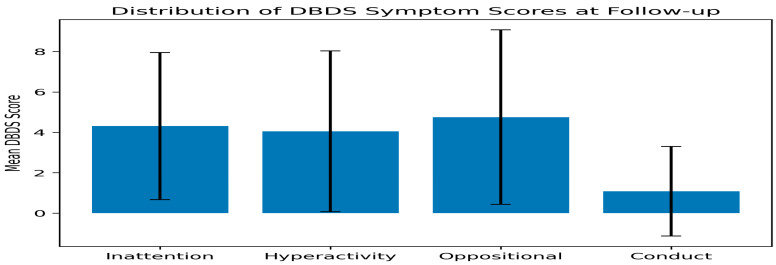
Distribution of attention–behavioral symptoms assessed using the Disruptive Behaviour Disorders Scale (DBDS) at follow-up.

**Figure 5:**
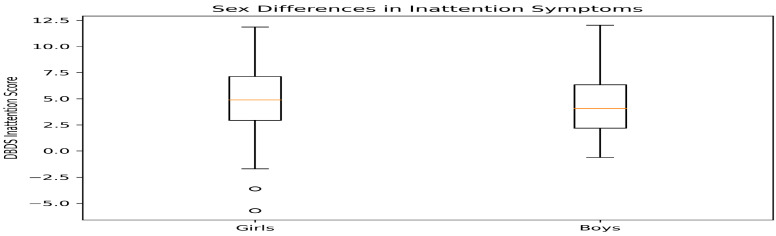
Distribution of Disruptive Behaviour Disorders Scale (DBDS) inattention scores according to sex. Boys demonstrated significantly higher inattention scores compared with girls (Mann–Whitney U test, *p* = 0.008). Empty circles represent outliers and orange lines indicate median values.

Figures were generated to illustrate patient flow, study timeline, HRQoL changes, and attention–behavioral outcomes (Figure 1, Figure 2, Figure 3, Figure 4 and Figure 5).

## 3. Results

### 3.1. Participant Characteristics

A total of 59 pediatric patients who underwent successful catheter ablation for symptomatic supraventricular tachycardia were included in the final analysis. The participant flow, including eligibility assessment and exclusions, is illustrated in the STROBE-compliant flow diagram (Figure 1).

The median age of the study population was 13 years (interquartile range: 10–15), with a mean age of 12.41 ± 3.12 years. The cohort included 34 girls (57.6%) and 25 boys (42.4%). Baseline demographic and clinical characteristics of the study population are summarized in Table 1.

The distribution of educational level among participants was as follows: kindergarten (1.7%), primary school (28.8%), middle school (27.1%), and high school (42.4%). Educational level did not differ significantly between sexes (χ^2^ test, *p* = 0.282). Review of procedural records showed that no acute complications occurred during or immediately after catheter ablation in the included cohort.

### 3.2. Psychiatric Diagnoses at Follow-Up

At the six-month follow-up assessment, 18 participants (30.5%; 95% confidence interval (CI), 20.3–43.1%) met diagnostic criteria for at least one psychiatric disorder according to the structured K-SADS-PL interview, whereas 41 participants (69.5%) had no psychiatric diagnosis. The distribution of psychiatric diagnoses identified at follow-up is presented in Table 2.

The most frequently observed diagnostic category was anxiety disorders, identified in 9 participants (15.3%; 95% CI, 8.2–26.5%). Depressive disorders were present in 4 participants (6.8%; 95% CI, 2.7–16.2%), adjustment disorder in 3 participants (5.1%; 95% CI, 1.7–13.9%), and attention-deficit/hyperactivity disorder in 2 participants (3.4%; 95% CI, 0.9–11.5%).

The prevalence of psychiatric diagnoses did not differ significantly between girls and boys (*p* = 1.00). However, participants with a psychiatric diagnosis were significantly older than those without a diagnosis (median age 14.0 vs. 12.0 years; *p* = 0.001).

### 3.3. Changes in Health-Related Quality of Life After Catheter Ablation

Health-related quality of life improved significantly across all PedsQL™ domains following catheter ablation (Table 3).

Median physical functioning scores increased from 47.5 (IQR: 32.5–62.5) before the procedure to 65.0 (IQR: 56.3–72.5) after ablation (*p* < 0.001). Emotional functioning scores increased from 32.5 (IQR: 25.0–45.0) to 42.5 (IQR: 35.0–47.5) (*p* < 0.001). Social functioning scores increased from 45.0 (IQR: 40.0–50.0) to 47.5 (IQR: 43.8–50.0) (*p* < 0.001). School functioning scores increased from 32.5 (IQR: 22.5–41.3) to 40.0 (IQR: 32.5–45.0) (*p* < 0.001).

These findings indicate significant improvements in patient-reported functioning across physical, emotional, social, and school-related domains following catheter ablation.

### 3.4. Attention–Behavioral Symptoms at Follow-Up

Attention and behavioral symptoms assessed using the Disruptive Behavior Disorders Scale (DBDS) were analyzed descriptively at the post-ablation follow-up visit (Table 4).

The mean inattention score was 4.31 ± 3.64 (median 3; IQR 2–6), and the mean hyperactivity score was 4.05 ± 3.98 (median 3; IQR 1–5). The mean oppositional defiant score was 4.75 ± 4.32 (median 4; IQR 2–6.5), and the mean conduct problem score was 1.08 ± 2.22 (median 0; IQR 0–1).

Because no pre-ablation DBDS data were available, these findings reflect the distribution of attention–behavioral symptoms at follow-up rather than changes attributable to the ablation procedure.

### 3.5. Sex Differences in Attention–Behavioral Symptoms

When DBDS scores were compared by sex (Table 5), boys demonstrated significantly higher inattention scores than girls (median 4 vs. 2; *p* = 0.008, r = 0.35), indicating a moderate effect size.

No statistically significant sex differences were observed for hyperactivity (*p* = 0.468), oppositional defiant symptoms (*p* = 0.556), or conduct-related problems (*p* = 0.314).

### 3.6. Sex Differences in HRQoL Change Scores

Changes in HRQoL scores before and after ablation were compared between girls and boys (Table 6). Improvements were observed in both sexes across all HRQoL domains. However, the magnitude of HRQoL change did not differ significantly between girls and boys in any domain (all *p* > 0.05). These findings suggest that the improvement in HRQoL following catheter ablation was similar for both sexes.

### 3.7. Associations Between Age and Attention–Behavioral Symptoms

Spearman correlation analyses showed that increasing age was significantly associated with higher inattention scores (r = 0.373, *p* = 0.004) and higher conduct problem scores (r = 0.301, *p* = 0.021).

No significant correlations were found between age and hyperactivity (*p* = 0.911) or oppositional defiant symptoms (*p* = 0.230).

### 3.8. Distribution of DBDS Cutoff Scores

Using predefined cutoff thresholds, 8 participants (13.6%) exceeded the cutoff for inattention symptoms (≥10), 6 participants (10.2%) exceeded the cutoff for hyperactivity symptoms (≥10), 16 participants (27.1%) exceeded the cutoff for oppositional defiant behaviors (≥6), and 6 participants (10.2%) exceeded the cutoff for conduct-related problems (≥6).

The proportions of participants exceeding these thresholds did not differ significantly between girls and boys for any DBDS subscale (all *p* > 0.05).

### 3.9. HRQoL Change According to Psychiatric Diagnosis

When changes in HRQoL scores were compared between participants with and without psychiatric diagnoses, improvements were observed in both groups across all domains.

Participants with psychiatric diagnoses showed a significantly greater improvement in emotional functioning compared with those without psychiatric diagnoses (*p* = 0.007). No statistically significant group differences were observed for physical functioning (*p* = 0.86), social functioning (*p* = 0.69), or school functioning (*p* = 0.50).

All statistical analyses were interpreted cautiously given the exploratory nature of the behavioral analyses and the absence of baseline behavioral data. 

## 4. Discussion

The present study evaluated changes in health-related quality of life (HRQoL) and attention–behavioral outcomes in children undergoing catheter ablation for symptomatic supraventricular tachycardia (SVT). The findings demonstrate significant improvements across all HRQoL domains following ablation, together with identifiable patterns in psychiatric diagnoses and attention–behavioral symptoms at follow-up. These results suggest that successful arrhythmia treatment may be associated with improvements in several aspects of daily functioning in pediatric patients with SVT.

### 4.1. HRQoL Improvements After Catheter Ablation

In the present cohort, HRQoL improved significantly across physical, emotional, social, and school functioning domains following catheter ablation. These findings are consistent with previous studies indicating that successful elimination of recurrent tachyarrhythmia episodes is associated with improved daily functioning and symptom relief in children with SVT [5,17].

Several mechanisms may explain these improvements. Recurrent tachycardia episodes are frequently associated with palpitations, fatigue, dizziness, exercise intolerance, and school absenteeism. Successful rhythm control may reduce these symptoms and allow children to participate more fully in normal physical, social, and academic activities.

In addition, uncertainty related to unpredictable arrhythmia episodes may generate significant emotional distress for both children and caregivers. The resolution of arrhythmia symptoms may therefore contribute not only to physical recovery but also to improvements in emotional well-being and overall psychosocial functioning.

These findings are consistent with contemporary pediatric electrophysiology literature reporting procedural success rates exceeding 90–95% with low complication rates and substantial reductions in symptom burden after catheter ablation [17,18]. Increasingly, outcome studies emphasize that the benefits of catheter ablation extend beyond rhythm control and include improvements in patient-reported outcomes and quality of life [19].

Importantly, improvements in HRQoL were observed in both girls and boys, and the magnitude of HRQoL change did not differ significantly between sexes. This suggests that the overall benefits of catheter ablation on patient-reported functioning appear to be similar across sex groups.

However, the observational design of the present study does not allow causal inference regarding HRQoL improvements. Because baseline HRQoL data were partly obtained retrospectively from caregiver recall, the observed improvements may also reflect regression to the mean, recall bias, or expectancy effects related to treatment.

### 4.2. Psychiatric Outcomes at Follow-Up

At follow-up, approximately one-third of participants met criteria for at least one psychiatric diagnosis (30.5%; 95% CI, 20.3–43.1), with anxiety-related disorders being the most common. This prevalence is broadly consistent with previous studies reporting increased rates of psychological symptoms among children and adolescents with chronic cardiac conditions compared with healthy peers or population norms [12,14,19].

Children with recurrent tachyarrhythmias may experience prolonged periods of uncertainty before definitive treatment, which may contribute to heightened anxiety, anticipatory stress, or maladaptive coping strategies. Even after successful medical or interventional treatment, psychological responses associated with chronic illness may persist.

Interestingly, children with psychiatric diagnoses in the present study demonstrated greater improvements in emotional functioning scores compared with those without psychiatric diagnoses. One possible explanation is that children with higher baseline emotional distress may experience greater perceived relief following successful arrhythmia treatment. However, because baseline psychiatric diagnostic data were not available, these findings should be interpreted cautiously.

Overall, these results highlight that while successful ablation may alleviate arrhythmia-related physical symptoms, psychological vulnerability may persist in a subset of patients. Therefore, psychosocial evaluation may represent an important component of comprehensive follow-up care in pediatric arrhythmia populations.

### 4.3. Attention–Behavioral Symptoms

Attention–behavioral symptoms assessed using the Disruptive Behavior Disorders Scale (DBDS) revealed several noteworthy patterns. Boys demonstrated significantly higher inattention scores than girls, consistent with epidemiological studies reporting higher prevalence of attention-related symptoms in boys during childhood and adolescence [20].

In addition, increasing age was associated with higher inattention and conduct-related symptom scores. Older children and adolescents may experience greater academic demands, social expectations, and emotional challenges, which could make attentional or behavioral difficulties more noticeable.

It is important to emphasize that attention–behavioral assessments in this study were conducted only at the post-ablation follow-up visit. Therefore, these findings describe the distribution of behavioral symptoms within the cohort rather than changes attributable to catheter ablation. No conclusions can be drawn regarding whether ablation improves, worsens, or has no effect on attention–behavioral functioning.

Nevertheless, the presence of subclinical behavioral symptoms in a subset of patients may indicate that children with chronic cardiac conditions could benefit from broader psychosocial screening during routine follow-up.

These post-ablation behavioral findings may reflect pre-existing developmental vulnerability, illness-related psychosocial burden, or age-related contextual demands rather than direct effects of the ablation procedure.

### 4.4. Clinical Implications

The findings of this study support a more comprehensive approach to outcome evaluation in pediatric electrophysiology. Traditionally, procedural success and arrhythmia elimination have been considered the primary indicators of treatment effectiveness. However, increasing attention is being paid to patient-centered outcomes such as quality of life and psychosocial functioning.

Routine assessment of HRQoL and basic psychological screening during follow-up may help clinicians identify children who could benefit from additional psychosocial support. A practical follow-up strategy may include brief HRQoL and behavioral screening at approximately 6–12 months after ablation to identify patients with persistent psychosocial difficulties.

Integrating cardiologic and psychosocial assessment may therefore improve long-term care in pediatric patients undergoing arrhythmia treatment.

### 4.5. Strengths

This study has several important strengths. First, it integrates cardiologic outcomes with structured psychiatric assessment and standardized HRQoL measurement, providing a multidimensional evaluation of recovery following pediatric catheter ablation. Second, psychiatric diagnoses were established using a structured clinical interview (K-SADS-PL) conducted by a child and adolescent psychiatrist, which increases diagnostic reliability. Third, the use of validated instruments for HRQoL and behavioral assessment strengthens the methodological robustness of the study.

### 4.6. Limitations

Several limitations should be considered when interpreting these findings.

First, because baseline HRQoL data were obtained retrospectively through caregiver recall, the possibility of recall bias should be considered when interpreting pre-post differences.

Second, an informant shift may have occurred in some cases because baseline HRQoL data could be caregiver-reported, whereas follow-up assessments for older children were self-reported. This methodological issue may influence the comparability of paired HRQoL measurements.

Third, psychiatric and attention–behavioral assessments were conducted only at the post-ablation follow-up visit, and no baseline behavioral data were available. Therefore, the study cannot determine whether behavioral symptoms changed following catheter ablation.

Fourth, the study was conducted at a single tertiary center with a moderate sample size, which may limit generalizability. In addition, analyses were restricted to patients with successful acute ablation, which may introduce selection bias by excluding patients with unsuccessful procedures or early recurrence. Moreover, several procedure-related variables, including arrhythmia mechanism subtype, ablation laterality, energy source distribution, fluoroscopy time, recurrence at follow-up, and antiarrhythmic medication use, were not systematically available for all participants, thereby limiting more detailed procedural subgroup analyses.

Finally, behavioral symptom assessments were based on parent-report questionnaires, which may be influenced by reporting bias.

### 4.7. Future Research

Future studies should further clarify the psychosocial recovery trajectory following arrhythmia treatment in pediatric populations. Prospective longitudinal studies including both pre- and post-procedural psychological assessments would provide more robust insight into the relationship between arrhythmia treatment and mental health outcomes.

Multicenter studies with larger sample sizes may help improve generalizability. In addition, future research should investigate potential predictors of psychosocial recovery, including arrhythmia burden, duration of symptoms before ablation, healthcare utilization, and family psychosocial factors.

## 5. Conclusions

In conclusion, catheter ablation in children with supraventricular tachycardia was associated with significant improvements in health-related quality of life across multiple domains. However, a meaningful proportion of patients continued to exhibit psychiatric or attention–behavioral symptoms at follow-up. These findings highlight the importance of integrating psychosocial assessment into the routine clinical follow-up of pediatric patients undergoing arrhythmia treatment.

## Figures and Tables

**Table 1:** Baseline demographic and clinical characteristics of the study population.

Variable	Value
Age, median (IQR)	13.0 (10.0–15.0)
Girls, *n* (%)	34 (57.6)
Boys, *n* (%)	25 (42.4)
Diagnosis	Supraventricular tachycardia
Structural heart disease	None
Prior cardiac surgery	None
Successful ablation	59 (100%)
Median follow-up duration (months)	6

**Table 2:** Psychiatric diagnoses at follow-up based on the Schedule for Affective Disorders and Schizophrenia for School-Age Children-Present and Lifetime Version (K-SADS-PL) interview.

Diagnosis	n (%)	95% CI
Any psychiatric diagnosis	18 (30.5)	20.3–43.1
Anxiety disorders	9 (15.3)	8.2–26.5
Depressive disorders	4 (6.8)	2.7–16.2
Adjustment disorder	3 (5.1)	1.7–13.9
Attention-deficit/hyperactivity disorder	2 (3.4)	0.9–11.5

**Table 3:** Changes in Pediatric Quality of Life Inventory (PedsQL™) domain scores before and after catheter ablation. Values are presented as median (interquartile range). Comparisons were performed using the Wilcoxon signed-rank test. Effect size r was calculated as |Z|/√N.

Domain	Pre-Ablation Median (IQR)	Post-Ablation Median (IQR)	Z	*p*	Effect Size (r)
Physical	47.5 (32.5–62.5)	65.0 (56.3–72.5)	−6.612	<0.001	0.861
Emotional	32.5 (25.0–45.0)	42.5 (35.0–47.5)	−6.461	<0.001	0.841
Social	45.0 (40.0–50.0)	47.5 (43.8–50.0)	−6.589	<0.001	0.858
School	32.5 (22.5–41.3)	40.0 (32.5–45.0)	−6.027	<0.001	0.785

**Table 4:** Post-Ablation Disruptive Behaviour Disorders Scale (DBDS) subscale scores.

Subscale	Mean ± SD	Median (IQR)
Inattention	4.31 ± 3.64	3 (2–6)
Hyperactivity	4.05 ± 3.98	3 (1–5)
Oppositional defiant	4.75 ± 4.32	4 (2–6.5)
Conduct problems	1.08 ± 2.22	0 (0–1)

**Table 5:** Sex differences in Disruptive Behaviour Disorders Scale (DBDS) subscale scores.

Subscale	Girls Mean ± SD	Boys Mean ± SD	*p*
Inattention	3.59 ± 3.64	5.28 ± 3.46	0.008
Hyperactivity	3.68 ± 3.76	4.56 ± 4.29	0.468
Oppositional	4.68 ± 3.94	4.84 ± 4.86	0.556
Conduct	0.85 ± 1.84	1.40 ± 2.65	0.314

**Table 6:** Sex-Stratified Comparison of health-related quality of life (HRQoL) Change Scores After Catheter Ablation.

HRQoL Domain	Girls Mean ± SD	Boys Mean ± SD	Girls Median (IQR)	Boys Median (IQR)	*p*-Value
Physical functioning	160.29 ± 127.79	166.00 ± 128.67	150.00 (75.00–250.00)	150.00 (50.00–300.00)	0.96
Emotional functioning	62.50 ± 78.64	80.44 ± 77.98	37.50 (0.00–118.75)	50.00 (25.00–125.00)	0.35
Social functioning	21.32 ± 53.35	28.00 ± 41.03	0.00 (0.00–25.00)	25.00 (0.00–50.00)	0.15
School functioning	34.56 ± 61.55	66.52 ± 88.15	0.00 (0.00–50.00)	50.00 (0.00–125.00)	0.13

Notes: Values represent change scores calculated as post-ablation minus pre-ablation HRQoL scores. Comparisons between girls and boys were performed using the Mann–Whitney U test. No statistically significant sex-based differences in HRQoL improvement were observed across any domain (all *p* > 0.05).

## Data Availability

The data presented in this study are available on reasonable request from the corresponding author. The data are not publicly available due to ethical and privacy considerations.

## Abbreviations

DBDS	DSM-IV Based Disruptive Behavior Disorders Screening and Assessment Scale
DSM-5	Diagnostic and Statistical Manual of Mental Disorders, Fifth Edition
HRQoL	health-related quality of life
K-SADS-PL	Schedule for Affective Disorders and Schizophrenia for School-Age Children—Present and Lifetime Version
PedsQL™	Pediatric Quality of Life Inventory
SVT	supraventricular tachycardia
